# Orchestrating mitochondria in neurons: Cytoskeleton as the conductor

**DOI:** 10.1002/cm.21585

**Published:** 2019-12-09

**Authors:** Carlos Cardanho‐Ramos, Andreia Faria‐Pereira, Vanessa Alexandra Morais

**Affiliations:** ^1^ Instituto de Medicina Molecular ‐ João Lobo Antunes, Faculdade de Medicina Universidade de Lisboa Lisbon Portugal

**Keywords:** docking, mitochondria, neuronal cytoskeleton, synapse, transport

## Abstract

Mitochondria are crucial to support synaptic activity, particularly through ATP production and Ca^2+^ homeostasis. This implies that mitochondria need to be well distributed throughout the different neuronal sub‐compartments. To achieve this, a tight and precise regulation of several neuronal cytoskeleton players is necessary to transport and dock mitochondria. As post‐mitotic cells, neurons are highly dependent on mitochondrial quality control mechanisms and several cytoskeleton proteins have been implicated in mitophagy. Therefore, all of these processes are orchestrated by the crosstalk between mitochondria and the neuronal cytoskeleton to form a coordinated and tuned symphony.

## 1 INTRODUCTION

The brain accounts for 20–25% of calorie intake and O_2_ consumption, mainly because neurons largely rely on oxidative phosphorylation for the production of ATP (Rolfe & Brown, 1997). Additionally, neurons require mitochondria for Ca^2+^ homeostasis and maintenance of their action potential. As sub‐compartmentalized cells, one can anticipate that neurons have different pools of mitochondria (Fedorovich, Waseem, & Puchkova, 2017). Therefore, in order to achieve this, a tight regulation of mitochondrial transport and turnover is expected. All of these processes are highly dependent on the neuronal cytoskeleton. Surprisingly, and despite the extreme importance these processes have, they have been massively overlooked, particularly at the level of the synapse.

In neurons, it is thought that most mitochondria are formed in the cell body and then need to travel to the other neuronal sub‐compartments, like dendrites and pre‐synaptic sites. Also, it is assumed that upon damage, mitochondria need to return to the cell body for consequent degradation and clearance. However, mitochondrial biogenesis (Amiri & Hollenbeck, 2008) and mitophagy (Ashrafi, Schlehe, LaVoie, & Schwarz, 2014) have also been reported to occur away from the cell body. Taking into account that in neurodegenerative diseases, synaptic dysfunction precedes neuronal loss and that the preponderance of mitochondria resides within axons and dendrites, one could speculate that efficient mitochondrial turnover is as relevant in distal compartments as in the soma. Nonetheless, this implies a tight regulation between mitochondrial transport and turnover. Indeed, neuronal mitochondria are very dynamic; they can travel back and forward, alternating between moving and stationary periods. Mitochondria can also fuse and divide. Dynamin‐related protein 1 (Drp1), mitochondrial fission protein 1 (Fis1) and mitochondrial fission factor are proteins involved in fission (Losón, Song, Chen, & Chan, 2013); whereas Mitofusin 1 and 2 (Mfn1 and Mfn2) and Optic Atrophy 1 (OPA‐1) are known to be involved in fusion of the outer and inner mitochondrial membranes, respectively (Song, Ghochani, McCaffery, Frey, & Chan, 2009). These dynamic processes are highly dependent on microtubules, but also on the actin cytoskeleton. Impressively, the disruption of mitochondrial transport (Sheng & Cai, 2012) and fission‐fusion cycles (Verstreken et al., 2005) in neurons leads to defects in neurotransmitter release, vesicle recycling and, ultimately, in synaptic plasticity. This tight regulation of mitochondrial transport and turnover is even more crucial when assessing synaptic function. Remarkably, one‐third of the synapse is occupied by mitochondria (Wilhelm et al., 2014). During synaptic activation by veratridine, a high‐energy demanding process, mitochondria are retained at presynaptic terminals and postsynaptic dendritic spines (D. T. W. Chang, Honick, & Reynolds, 2006). Moreover, KCl‐induced depolarization, as well as electrical stimulation, recruits mitochondria to dendrites (D. T. W. Chang et al., 2006). Additionally, an increased number of stationary mitochondria at synapse have been observed upon activation of Ca^2+^ (Ohno et al., 2011) and Na^+^/K^+^ ATPase channels (Zhang, Ho, Kintner, Sun, & Chiu, 2010). Bearing all of these in mind, we speculate that mitochondria are anchored at synapses, where high levels of ATP and Ca^2+^ buffering are required. However, the molecular mechanisms that enable mitochondria to sense when to move or halt are still elusive. Understanding the role of the cytoskeleton, and its respective motor proteins, in mitochondrial transport and turnover is key to define how this organelle is involved in maintaining a functional neuron, and to determine whether neurodegeneration occurs because these mitochondrial processes go astray.

## 2 MITOCHONDRIAL TRANSPORT

### 2.1 How are mitochondria transported antero and retrogradely in neurons?

**Figure 1:**
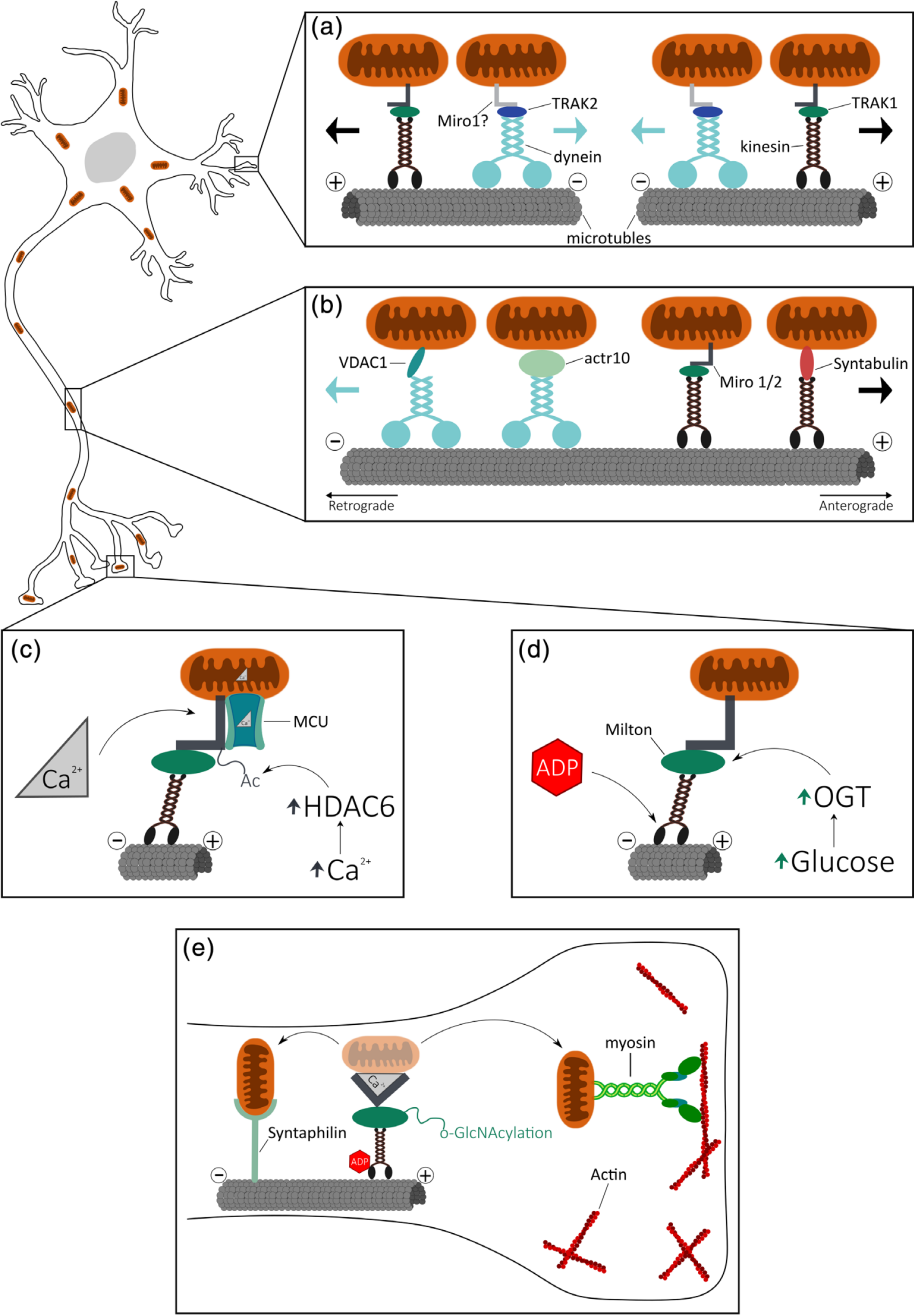
Mechanisms of mitochondrial transport and docking in mammalian neurons (a) Dendritic transport. Dendritic microtubules have mixed polarity; therefore, dynein and kinesin undergo anterograde or retrograde transport. Although, in dendrites, mitochondrial transport is mainly mediated by dynein, TRAK2, and possibly Miro1; kinesin, TRAK1, and Miro2 may also be involved. (b) Axonal transport. Axonal microtubules have their minus‐ends directed to the cell body and the plus‐ends toward the periphery. Kinesin is responsible for the anterograde transport, mediated by Syntabulin, TRAK1 and possibly Miro2. Dynein is responsible for retrograde transport, mediated by the dynactin subunit, actr10 or an unknown adaptor, possibly VDAC1. (c) Mitochondrial docking dependent on Ca^2+^. Miro is able to sense Ca^2+^ levels, promoting entry of Ca^2+^ to mitochondria through the MCU, which leads to conformational changes in Miro and disruption of the Miro‐MCU complex. Elevated Ca^2+^ activates HDAC6, leading to deacetylation of Miro. Together, these mechanisms promote the detachment of mitochondria from kinesin. (d) Mitochondrial docking dependent on ADP and Glucose. Synapses are regions with high ATP consumption and increased levels of ADP. ADP can bind to kinesin inhibiting its motor function. Synaptic activity also promotes entry of glucose, which activates O‐GlcNAc transferase (OGT), leading to O‐GlcNAcylation of Milton. Together, these mechanisms promote the detachment of mitochondria from kinesin. (e) Mitochondrial anchoring at synapse. When mitochondria detach from kinesin, they can either undergo microtubule‐dependent docking, mediated by Syntaphilin; or actin‐dependent docking, through a myosin

Microtubules are important to determine cell polarity and neurons are a striking example for this. Both axons and dendrites are supported by stable microtubules that together with the neuro‐ and microfilaments form the neuronal cytoskeleton (Fletcher & Mullins, 2010). In mammals, microtubules in axons and dendrites are organized differently. Axonal microtubules have their minus ends directed toward the cell body, while their plus ends are directed toward the periphery. In contrast, dendritic microtubules have a mixed polarity (Figure 1a,b). The actin cytoskeleton, a dynamic network made up of actin polymers and associated actin binding proteins, can essentially form two different structures: actin‐rings and actin patches. Cortical actin, together with spectrin, can be organized in ring‐like structures that wrap around neuronal processes to give mechanical support and to organize important membrane proteins, such as sodium channels (Xu, Zhong, & Zhuang, 2013). Actin patches are areas enriched in branched actin that play a role in anchoring vesicles and organelles at the base of spines (Bommel, Konietzny, Kobler, Bär, & Mikhaylova, 2019). Longitudinal actin fibers have also been observed in neurons, but the function of these long bundles is still unclear.

While, disruption of microtubules in neurons, using nocodazole, leads to the increase of stationary mitochondria (Ligon & Steward, 2000; Morris & Hollenbeck, 1995) and the accumulation of perinuclear mitochondria (Tanaka et al., 1998), impairing actin assembly increases mitochondrial transport (Chada & Hollenbeck, 2004; Gutnick, Banghart, West, & Schwarz, 2019; Morris & Hollenbeck, 1995). These observations suggest that mitochondrial transport along axons is highly influenced by microtubules' stability whereas, once mitochondria arrive at the synapse, actin becomes the major cytoskeleton player governing mitochondrial arrest.

#### 2.1.1 Anterograde transport

Anterograde transport is characterized by the movement from the cell body to the synapse (or cell periphery). Kinesins are the motor proteins that “walk” toward the plus‐end of microtubules, being responsible for the transport of cargo to the synapse (Figure 1b). These proteins contain two heavy chain N‐terminal motor domains (KHC), connected to two light chain C‐terminal cargo‐binding domains (KLC). Mutations in the cargo‐binding domain or in the motor‐domain of kinesin‐1 reduce the anterograde transport of mitochondria in *Drosophila* larvae axons (Pilling, Horiuchi, Lively, & Saxton, 2006). Overexpression of kinesin‐1 cargo‐binding domain also decreases mitochondrial anterograde transport in rat hippocampal neurons (Cai, Gerwin, & Sheng, 2005). Therefore, mitochondria are essentially transported by the kinesin superfamily of proteins (KIF), namely the kinesin‐1 (Pilling et al., 2006; Tanaka et al., 1998). The kinesin‐1 family is composed of three proteins: KIF5A and KIF5C, only expressed in neurons; and KIF5B which is ubiquitously expressed (Xia, Rahman, Yang, & Goldstein, 1998). Despite the striking effect in mitochondrial transport, it is unlikely that these motor proteins are able to bind directly to mitochondria. Therefore, identifying the adaptor proteins that bind to mitochondria is of key relevance to understand how mitochondrial transport is regulated in neurons.

Milton was first identified in *Drosophila* as an adaptor protein that links mitochondria to kinesin‐1 (Stowers, Megeath, Górska‐Andrzejak, Meinertzhagen, & Schwarz, 2002). The N‐terminal of Milton interacts with the C‐terminal of KHC to transport mitochondria along axons, reaching synaptic terminals. In accordance with this, expressing Milton‐null mutants increased mitochondria in the cell body (Glater, Megeath, Stowers, & Schwarz, 2006). However, Milton does not have a clear mitochondrial binding site therefore it requires another protein to recruit mitochondria to kinesin‐1. The Mitochondria Rho GTPase (Miro) localizes to the outer mitochondrial membrane (OMM) through its C‐terminal transmembrane domain and has also been implicated in mitochondrial transport (Fransson, Ruusala, & Aspenström, 2003). In motor neuron axons of *Drosophila* larvae, loss of Miro altered both anterograde and retrograde transport (Russo et al., 2009). Moreover, expression of Miro mutants led to an increase in perinuclear mitochondria and reduced the number of mitochondria at neuromuscular junctions (NMJs) (Guo et al., 2005). Interestingly, it has been observed that Milton interacts with Miro (Glater et al., 2006) to form a complex capable of mediating the connection of mitochondria to kinesin‐1 and promoting anterograde mitochondria transport in neurons.

In mammals, Trafficking Kinesin Protein (TRAK) 1 and TRAK2 are two Milton orthologues; and Miro1 and Miro2 are two Miro orthologues. As in *Drosophila*, these also form a complex that connect mitochondria to kinesin‐1 and are required for axonal transport (MacAskill, Brickley, Stephenson, & Kittler, 2009). However, it seems that different orthologues may have different functions. Both TRAK1 and TRAK2 are associated with mitochondria, being TRAK1 mainly localized in axons, whereas TRAK2 is more present in dendrites (van Spronsen et al., 2013) (Figure 1a,b). Accordingly, knockdown of TRAK1, but not TRAK2, impairs axonal mitochondrial transport (Brickley & Stephenson, 2011), whereas, knockdown of TRAK2 alters transport in dendrites (van Spronsen et al., 2013). This can be explained by a difference in the conformation of these two proteins. While TRAK1 has a dynamic conformation, enabling the interaction with kinesin‐1 and dynein (Figure 1b), a motor protein responsible for retrograde transport; TRAK2 forms an interaction between its N‐terminal and C‐terminal domains and appears to preferentially bind to dynein (Figure 1a). Remarkably, inducing a head‐to‐tail interaction in TRAK1 forces its translocation to dendrites (van Spronsen et al., 2013). Thus, in mammals the mitochondria transport machinery seems more complex and specialized, because the type of movement is specifically dependent on the type of TRAK protein that is bound to Miro. TRAK1 is more related to anterograde transport (Figure 1b) and TRAK2 is associated with retrograde transport (Figure 1a). In mouse embryonic fibroblasts (MEFs) it has been observed that the TRAK2 retrograde movement is dependent on Miro1 (López‐Doménech et al., 2018), which lead us to speculate that different Miros might also have different functions. Additionally, Miro1/2 double knock out cells still show some mitochondrial transport (López‐Doménech et al., 2018). This implies that there are other players involved in the connection of mitochondria to kinesin‐1. Fasciculation and elongation protein zeta‐1 is a brain specific protein that interacts with KIF5B to promote mitochondrial anterograde transport in hippocampal neurites; in a process crucial for the establishment of neuronal polarity (Ikuta et al., 2007). Although this transport mechanism is very important during developmental stages, it probably plays a minor role in mature neurons. Ran‐binding protein 2 (RanBP2) has also been proposed as an adaptor protein in mitochondrial transport (Cho et al., 2007); however this has never been shown in neurons. Mfn2 also interacts with Miro1/2 and TRAK1/2 (Misko, Jiang, Wegorzewska, Milbrandt, & Baloh, 2010). Neurons expressing Mfn2 mutants (Baloh, Schmidt, Pestronk, & Milbrandt, 2007) or lacking Mfn2 expression (Misko et al., 2010) have reduced transport and increased stationary mitochondria, in a mechanism that is independent of fusion. This places Mfn2 as a possible player involved in mitochondrial trafficking.

Syntabulin is an adaptor protein that contains a kinesin‐binding domain (KBD). Recently, it has also been observed that the C‐terminal domain of Syntabulin is able to associate with membranes, including the OMM (Cai et al., 2005). In neurons, downregulation of Syntabulin reduces the anterograde, but not retrograde, transport of mitochondria. Additionally, overexpression of the Syntabulin‐KBD disrupts the connection between mitochondria and kinesin‐1, reducing anterograde transport and also decreasing mitochondrial density along axonal processes (Cai et al., 2005). Thus, Syntabulin‐mediated anterograde trafficking appears as a novel mechanism to transport mitochondria in axons, independent of the Miro/TRAK complex (Figure 1b).

#### 2.1.2 Retrograde transport

In neurons, retrograde transport is characterized by the transport from the synapse to the cell body. This type of movement is mediated by dynein, a motor protein capable of “walking” toward the minus ends of microtubules (Figure 1b). Mutations in the Dynein heavy chains (motor domain) led to a reduction of retrograde transport of mitochondria, including decreased run length and duration (Pilling et al., 2006). Additionally, the dynein inhibitor ciliobrevin D prevents retrograde movement of mitochondria, but also affects the anterograde transport in axons of embryonic chicken dorsal root ganglion neurons (Sainath & Gallo, 2015). Dynactin is also involved in retrograde transport as it facilitates dynein processivity (Schroer, 2004), likewise it plays a role in anchoring dynein at microtubule plus ends (Moughamian & Holzbaur, 2012). Accordingly, dominant negative forms of dynactin are associated with altered mitochondrial movement in axons (Pilling et al., 2006). Additionally, dendritic mitochondrial transport is strongly affected if dynein and dynactin, but not kinesin‐1, are blocked (van Spronsen et al., 2013). Dynein and dynactin are, this way, not only responsible for mitochondrial retrograde transport, but also play an important role in dendritic mitochondrial transport (Figure 1a,b). However, how they bind to mitochondria is still unclear. In HEK293 cells, it has been demonstrated that dynein is able to bind to TRAK (van Spronsen et al., 2013), whereas Miro1 interacts with both dynein and dynactin (Morlino et al., 2014). In addition, loss of Miro also impaired retrograde movement (Russo et al., 2009). Nonetheless, there are probably other proteins more specifically involved in retrograde transport.

An interesting study in zebrafish has identified actr10, a dynactin subunit, to be involved in the retrograde transport of mitochondria in axons (Drerup, Herbert, Monk, & Nechiporuk, 2017). Actr10 mutants led to an accumulation of mitochondria in axon terminals and reduced retrograde, but not anterograde, transport (Figure 1b). This altered transport seems to be specific for mitochondria, as the movement of other organelles, such as lysosomes, was not affected (Drerup et al., 2017). Moreover, actr10 is able to bind to mitochondria independently of dynein and dynactin. This subunit does not have a transmembrane or membrane‐associated domain, but it can interact with the GDP‐bound forms of Drp1 (Drerup et al., 2017). These findings could suggest that mitochondrial transport machinery is not fully independent of the fission/fusion machinery.

Schwarzer and colleagues have shown that, in HeLa cells, VDAC1, an anion channel present on the OMM, is capable of interacting with the DYNLT1, a dynein light chain protein (Schwarzer, Barnikol‐Watanabe, Thinnes, & Hilschmann, 2002). Therefore, VDAC1 is also a possible candidate to link mitochondria to dynein (Figure 1b). However, further studies on the impact of VDAC1 on retrograde mitochondrial trafficking need to be performed.

### 2.2 How are mitochondria docked at the synapse?

Mitochondria are very dynamic organelles, however, as neurons differentiate and maturate the percentage of motile mitochondria decreases (Lewis, Turi, Kwon, Losonczy, & Polleux, 2016). It is estimated that, in neurons, around 70% of mitochondria are stationary, whereas the other 30% are motile. Since ATP has a very low diffusion rate (Hubley, Locke, & Moerland, 1996), it is crucial that mitochondria are able to produce energy in regions with high ATP demands. Moreover, the capacity of mitochondria to accumulate Ca^2+^ makes them one of the most important organelles to be present at synapse. Consequently, mitochondria not only have to be transported, they also require specific docking mechanisms, which have to be tightly regulated to respond rapidly to synaptic activity.

#### 2.2.1 Microtubule‐docking

Microtubules are usually associated with mitochondrial transport, but the recent involvement of Syntaphilin in this pathway changed this view (Figure 1e). Syntaphilin is a mitochondrial docking protein that is mainly localized in axons (Kang et al., 2008). It has been observed that mitochondria that co‐localize with Syntaphilin are immobile. Accordingly, *snph* null mice have increased mitochondrial transport in axons, but not in dendrites, confirming the specificity of Syntaphilin for axonal mitochondrial anchoring (Kang et al., 2008).

Syntaphilin is able to bind to mitochondria through its C‐terminal tail, which is moderately hydrophobic and therefore may interact directly with the OMM. Additionally, it also has a microtubule‐binding domain, which is responsible for keeping mitochondria docked at microtubules (Kang et al., 2008). Upon Ca^2+^ or electrical stimulation, the reduced mitochondrial transport was only observed in WT and not in *snph* null animals (Chen & Sheng, 2013), indicating that this docking mechanism is responsible for maintaining mitochondria in the vicinity of presynaptic terminals. Nevertheless, docking mechanisms need to be coordinated with motor proteins. The dynein light chain LC8 is crucial to stabilize the microtubule‐binding domain of Syntaphilin, facilitating its anchoring (Chen, Gerwin, & Sheng, 2009). Additionally, it has been observed that Syntaphilin has a KBD and this interaction is responsible for reducing ATPase activity of these motor proteins (Chen & Sheng, 2013). This led Chen and Sheng to formulate the “Engine‐switch and Brake” hypothesis, where Syntaphilin not only functions as a brake for mitochondria, but it also switches kinesin‐1 from the Miro‐TRAK complex, further enhancing mitochondrial docking (Chen & Sheng, 2013). It is still not clear which are the signals that turn the docking on and off, but Syntaphilin has several phosphorylation sites, making these plausible targets. Curiously, LKB1 and NUAK1 are two kinases involved in mitochondrial docking in axons, as loss of either proteins lead to an increase in mitochondrial transport. Overexpression of Syntaphilin can rescue these effects, indicating that Syntaphilin acts as a downstream target of these kinases (Courchet et al., 2013).

#### 2.2.2 Actin‐docking

Although axonal transport of mitochondria is mainly performed using microtubules, when microtubule assembly is disrupted in neurons treated with nocodazole (Ligon & Steward, 2000) or vinblastine (Morris & Hollenbeck, 1995), small mitochondrial movements still persist. Thus, suggesting that mitochondria can also be transported on actin cables. However, this has never been clearly observed in neurons. Additionally, no motor protein related with actin has been identified to be responsible for mitochondrial transport in neurons.

Actin is enriched at synapse, where it modulates synaptic morphology and, consequently, synaptic plasticity. Curiously, Wiskott–Aldrich syndrome protein‐family verprolin‐homologous protein (WAVE1), which is involved in actin polymerization, is required for mitochondria to enter dendritic spines (Sung et al., 2008). It has also been observed that disruption of the actin cytoskeleton actually increased the velocity of mitochondrial movement (Ligon & Steward, 2000; Morris & Hollenbeck, 1995) and that actin is required for mitochondrial docking along axons (Chada & Hollenbeck, 2004) and at presynapses (Gutnick et al., 2019). These observations pose an interesting view where mitochondria require microtubules to be transported, but once they get to regions with high ATP and Ca^2+^ buffering demands, such as synapses, they require the actin cytoskeleton for anchoring (Figure 1e).

One strong candidate protein to anchor mitochondria on actin cables is myosin‐XIX. Although it is the most widely studied myosin associated with mitochondria, its role in neurons is not fully known. Myosin‐XIX is able to bind to the OMM through its lipid‐binding domain (Shneyer, Ušaj, & Henn, 2015), but most importantly it also binds to Miro (Oeding et al., 2018). Indeed, in HeLa cells, expressing different myosin‐XIX mutants with an intact Miro‐binding is sufficient to disrupt kinesin and dynein from mitochondria, leading to an accumulation of perinuclear mitochondria (Oeding et al., 2018). Further studies in neurons are required to verify if myosin‐XIX can also anchor mitochondria at synapse. Another two interesting candidates are myosin‐V and myosin‐VI. They have recently been identified as possible anchor proteins that link mitochondria to actin cables in *Drosophila* axons. Loss of myosin‐V led to an increase in almost all parameters of mitochondrial transport in both directions, whereas loss of myosin‐VI only led to an increase of the retrograde transport. Deletion of either myosins reduced the time mitochondria spent immobile (Pathak, Sepp, & Hollenbeck, 2010). How these proteins interact with mitochondria is still elusive, hence it would be interesting to see if they can also interact with the Miro‐TRAK complex. Understanding these mechanisms will allow us to manipulate mitochondrial docking and stimulate synaptic activity.

### 2.3 What makes mitochondria stay at the synapse: Should they stay or should they go?

Upon synaptic stimulation, mitochondria are retained in regions with high ATP demands and Ca^2+^ pools. This docking may be dependent on microtubules and Syntaphilin (Kang et al., 2008) or on actin and myosin (Pathak et al., 2010). Intriguingly, how mitochondria know when to be transported or anchored and, how ATP and Ca^2+^ interfere with motor and adaptor proteins to dictate if mitochondria should “stay or go” remains to be clarified.

#### 2.3.1 ATP

Proper synaptic function requires high ATP levels for many processes, such as Na^+^/K^+^ ATPase channels and neurotransmitter vesicle recycling. Since ATP has a low diffusion rate, this high energy demand at synapse must be achieved through local ATP production. It has recently been observed that electrical stimulation drives ATP synthesis through glycolysis and oxidative phosphorylation (Rangaraju, Calloway, & Ryan, 2014). Therefore, mitochondria must be able to sense such compartments and have mechanisms that promote docking instead of transport.

Synaptic stimulation promotes ATP synthesis at presynaptic terminals. However, this is only possible if all the substrates are available at synapse. To achieve this, neurons, during synaptic activity, upregulate their surface glucose transporters and, hence, their glucose levels. Curiously, an increase in glucose levels reduces axonal mitochondria transport in both directions (Pekkurnaz, Trinidad, Wang, Kong, & Schwarz, 2014). OGT (O‐linked β‐N‐acetylglucosamine transferase or O‐GlcNAc transferase) is an enzyme responsible for the addition of a single sugar moiety to Milton, in a process called O‐GlcNAcylation. Glucose promotes the activity of OGT leading to an increase in Milton O‐GlcNAcylation, which in turn inhibits mitochondrial transport and promotes docking in axons (Pekkurnaz et al., 2014) (Figure 1d).

Mitochondria move slower in regions near synapse and their transport is inhibited if neurons are stimulated with glutamate (Mironov, 2007). Curiously, when neurons are co‐treated with glutamate and ATP, the activity of Na^+^/K^+^ ATPase channels is fully restored; nevertheless, mitochondrial transport in dendrites remains suppressed. Thus, ATP is not the energy sensor that forces mitochondria to stop at synapses. In contrast, injections of ADP decreased mitochondrial dendritic transport in the same extent as glutamate (Mironov, 2007). Since synaptic terminals are regions with elevated ATP consumption, they also have increased ADP levels. Once motor proteins require ATP to transport mitochondria, one could speculate that during synaptic stimulation ADP is more frequently rebound to kinesin and dynein, inhibiting their action and reducing mitochondrial transport near synapse (Figure 1d).

Alterations in glucose and ADP gradients can, therefore, slow down mitochondrial transport favoring their docking at synapse (Figure 1d).

#### 2.3.2 Ca^2+^

Mitochondria maintain a proper Ca^2+^ homeostasis at both presynaptic and postsynaptic terminals. To achieve this, substantial amounts of Ca^2+^ are sequestered through the mitochondrial calcium uniporter (MCU) (Billups & Forsythe, 2002). Therefore, it comes with no surprise that during synaptic transmission mitochondria accumulate near synapses, and that this docking is dependent on Ca^2+^ (D. T. W. Chang et al., 2006). Although Ca^2+^ can also influence actin and microtubules stabilization, there must be an additional mechanism able to more efficiently control mitochondrial docking at synapse.

Miro is located at the OMM and has two Ca^2+^‐binding EF hands (Fransson et al., 2003), placing it as a potential Ca^2+^ sensor and a regulator of mitochondrial transport. Accordingly, many authors have observed that neurons expressing Miro mutants lacking the two EF‐hands (Miro‐ΔEF) are no longer capable of arresting mitochondria in a Ca^2+^‐dependent manner both in axons (Wang & Schwarz, 2009) and in dendrites (MacAskill et al., 2009). However, there are two opposing views related to the mechanism through which Miro mediates mitochondrial docking. Wang et al. observed that increased Ca^2+^ maintained the complex kinesin‐Milton‐Miro together; therefore stationary mitochondria do not require the dissociation of kinesin from mitochondria. Instead, Ca^2+^ bound to Miro induces a conformational change that promotes the interaction with the *N*‐terminal motor domain of kinesin, thus displacing the motor protein from microtubules (Wang & Schwarz, 2009). In contrast, MacAskill et al. reported that Miro can bind directly to kinesin and this interaction is disrupted by Ca^2+^, forcing the detachment of kinesin from mitochondria (MacAskill, Rinholm, et al., 2009). The discrepancy in these findings can be explained by the fact that these observation were obtained from different neuronal compartments: axons versus dendrites. Nevertheless, and despite the opposing views, both mechanisms are mediated by Miro EF‐hands. Interestingly, they also observed a Ca^2+^‐dependent arrest of mitochondria moving in the retrograde direction, making it plausible that dynein is also capable of interacting with Miro in a similar way as kinesin. These mechanisms point out cytoplasmic Ca^2+^ as one of the main regulator of mitochondrial docking. In contrast, a recent study reported that mitochondrial matrix Ca^2+^ is an active modulator of mitochondrial transport in axons (K. T. Chang, Niescier, & Min, 2011). In this study, Chang et al. observed that blocking the MCU delayed the Ca^2+^‐dependent immobilization of mitochondria, despite increasing cytoplasmic Ca^2+^. Additionally, activating the MCU immediately abolished mitochondrial movement, without affecting cytoplasmic Ca^2+^ (K. T. Chang et al., 2011). Miro interacts with the MCU, and the import of Ca^2+^ into the mitochondria is dependent on the disruption of this complex (Niescier, Hong, Park, & Min, 2018) and on the EF‐hands of Miro (K. T. Chang et al., 2011). Curiously, mitochondrial transport requires an intact Miro‐MCU complex (Niescier et al., 2018). Altogether these observations lead us to develop a model where the Miro‐MCU complex modulate mitochondrial transport along the axon, but when they get to the synapse, the high levels of Ca^2+^ in this region forces the disruption of the Miro‐MCU complex along with conformational changes in Miro's EF‐hands. These changes promote mitochondrial Ca^2+^ import through the MCU and mitochondrial docking via Miro (Figure 1c). Thus, Miro plays a dual role in Ca^2+^ sensing and in mitochondrial transport.

Recently, a different mechanism through which Miro modulates mitochondrial transport in axons has been revealed. Histone deacetylase 6 deacetylates Miro and reduces mitochondrial transport, in a process dependent on Ca^2+^ and Miro's EF‐hands (Figure 1c) (Kalinski et al., 2019). Therefore, other post‐translational modifications of Miro may be required to modulate mitochondrial transport in neurons. All of these observations have focused on the link between mitochondria and kinesin, while a possible role of anchor proteins has been overlooked. Curiously, Syntaphilin‐mediated docking of mitochondria on microtubules is promoted by increased Ca^2+^ levels (Chen & Sheng, 2013). Whether or not the Miro‐MCU complex is involved in Syntaphilin anchoring is still unclear. Additionally, both myosin‐V and myosin‐VI have calmodulin‐binding domains and their processitivity is mediated by Ca^2+^. Thus, it would be worthwhile to understand how Ca^2+^ impacts the binding of myosins to mitochondria. One could speculate that Ca^2+^‐mediated conformational changes of Miro force the binding of Miro to myosins promoting mitochondrial anchoring at the synapse. Future studies are required to confirm these hypotheses.

### 2.4 What happens if mitochondrial transport and docking mechanisms are impaired?

Proper synaptic activity requires mitochondria for local production of ATP (Rangaraju et al., 2014) and Ca^2+^ buffering (Billups & Forsythe, 2002). Thus, disruption of the transport can lead to impaired synaptic transmission. In *Drosophila*, loss of Miro leads to a reduction of mitochondria at NMJs and, consequently, to a deficient neurotransmitter release during prolonged stimulation (Guo et al., 2005). Additionally, Miro1 null mice have decreased mitochondria in distal dendrites, but not in axons. This impaired dendritic mitochondrial positioning causes a marked decrease in dendritic complexity that accelerates neurodegeneration (López‐Doménech et al., 2016). Similarly, expression of mutant forms of Milton (Stowers et al., 2002) or loss of Syntabulin (Ma, Cai, Lu, Sheng, & Mochida, 2009) also leads to reduced number of mitochondria at neuronal processes, thus impairing synaptic activity. Syntaphilin null mice have reduced stationary mitochondria serving as local energy and Ca^2+^ buffering stations. Although this does not affect basal synaptic transmission, it increases Ca^2+^ levels at presynaptic terminals which results in enhanced short‐term facilitation during prolonged stimulation (Kang et al., 2008).

## 3 MITOCHONDRIAL TURNOVER: THEIR LIFE CYCLE?

The cell body is the main regulator of most neuronal functions. Therefore, it is assumed that most mitochondria are “born” there, then they travel to the synapse to exert their functions, but once they get damaged they need to travel back to the cell body to “die.” However, recent reports have observed that most of the machineries that allow mitochondrial turnover are also present at the synapse, such as lysosomes (Maday, Wallace, & Holzbaur, 2012). Accordingly, it has been observed that both mitochondrial biogenesis (Amiri & Hollenbeck, 2008) and mitochondrial degradation (Ashrafi et al., 2014) can occur away from the cell body. These results are still somewhat controversial and further studies are required to clarify these different views. Nonetheless, a good coordination between mitochondrial transport and turnover must be in place for proper neuronal function.

### 3.1 Mitophagy

Mitochondria are capable of sensing their own health and undergo a selective clearance pathway, known as mitophagy. When mitochondria are unhealthy, the mitochondrial kinase PINK1 accumulates at the OMM leading to the recruitment of Parkin, an E3‐ubiquitin ligase that, when phosphorylated, ubiquitinates several OMM proteins, ultimately targeting mitochondria for degradation (Leites & Morais, 2018; Narendra et al., 2010). This mechanism is well characterized in cell lines; however, initial reports using the same experimental setup in neurons were unable to detect mitophagy (van Laar et al., 2011). Nonetheless, recently it has been observed that PINK1/Parkin‐mediated mitophagy indeed occurs in neurons (Ashrafi et al., 2014; Cai, Zakaria, Simone, & Sheng, 2012). What is still unclear is where in the neuron this clearance process occurs.

**Figure 2:**
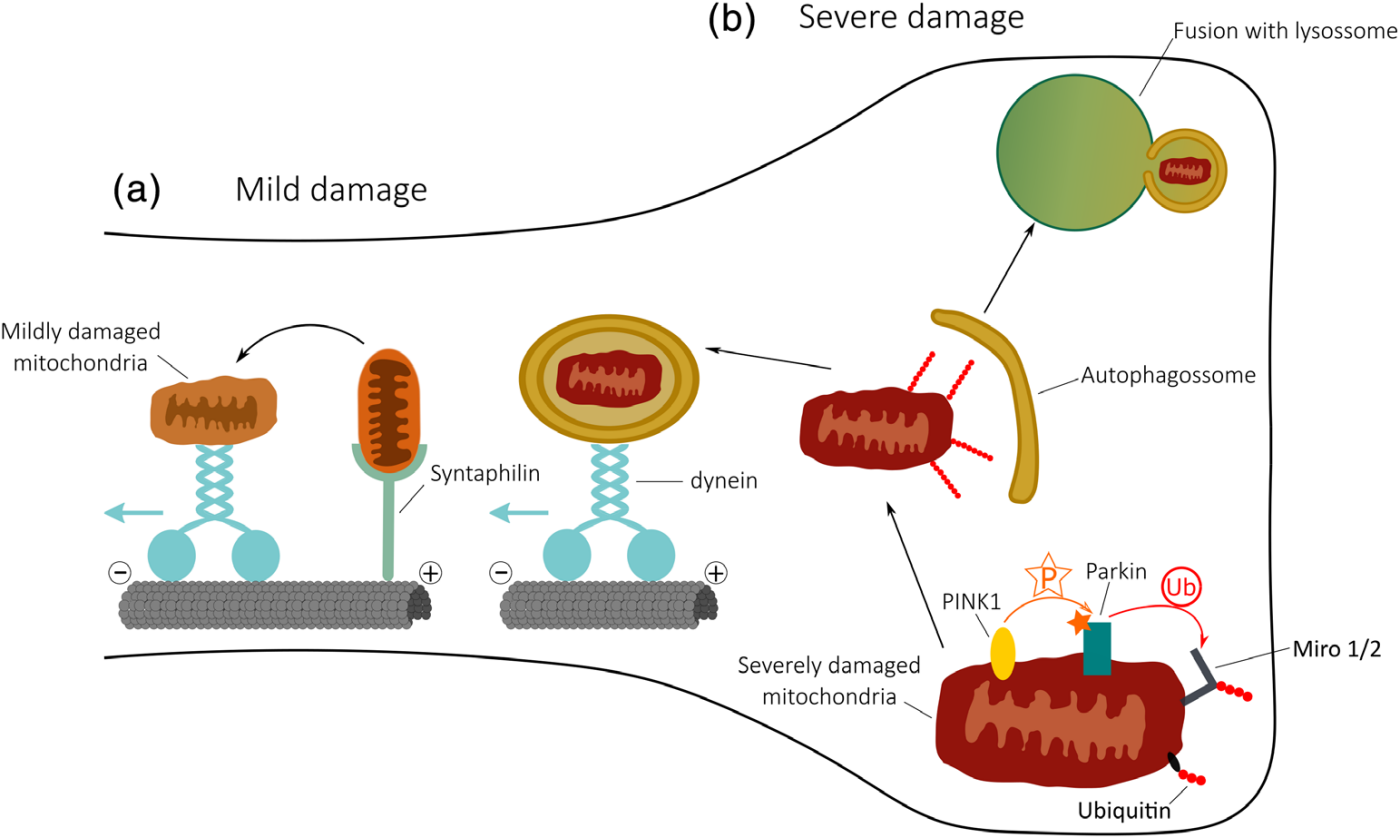
How neurons deal with distally damaged mitochondria: Mild versus severe damage(a) Upon mild damage, mitochondria in the vicinity of synapses detach from Syntaphilin and are transported retrogradely to the cell body for degradation. (b) Upon severe damage, mitochondria trigger local mitophagy, through the PINK1/Parkin pathway. When mitochondria are unhealthy, PINK1 accumulates on the mitochondrial membrane (OMM) and phosphorylates Parkin, which in turn ubiquitinates several mitochondrial substrates, including Miro. Ubiquitinated mitochondria are engulfed by autophagosomes, which can either undergo retrograde transport or local fusion with lysosomes

Cai et al. observed that depolarization of mitochondria induces Parkin translocation and a subsequent recruitment of LC3 and LAMP1 in the somatodendritic region. This was achieved by an increase in the mitochondrial retrograde transport (Cai et al., 2012). However, it has been observed that PINK1 and Parkin are capable of arresting mitochondria through degradation of Miro (Wang et al., 2011). This mitochondrial arrest also occurs when mitophagy is induced, leading us to wonder how damaged mitochondria are able to return to the cell body. One hypothesis is that mitochondria are engulfed into distal autophagosomes and then travel to the cell body using a Miro‐independent mechanism (Figure 2b). Accordingly, the formation of autophagosomes incorporating mitochondria has been observed in distal axons (Maday et al., 2012). Another interesting hypothesis was proposed by Ashrafi et al., in which depolarization of mitochondria promote PINK1/Parkin‐mediated degradation of Miro and distal mitophagy. They observed damaged mitochondria located away from the cell body, able to recruit LC3 and LAMP1, and consequently local degradation of mitochondria occurred (Figure 2b) (Ashrafi et al., 2014). Instead of opposing views, these are probably two complementary mechanisms. While most mitophagy probably occurs in the cell body, this is not a cell body exclusive event, and neurons are capable of degrading mitochondria distally as well (Figure 2b).

In order to maintain a healthy pool of mitochondria, damaged mitochondria do not only undergo mitophagy. Under mild damage, they can form mitochondria derived vesicles (MDV's), which enables mitochondria to remove intrinsic damage sections without degradation of the entire organelle (Sugiura, McLelland, Fon, & McBride, 2014). Interestingly, the formation of vesicles similar to MDV's was recently observed in neurons in response to pathological stress, and this process is dependent on Syntaphilin (Lin et al., 2017). Additionally, under mild damage, depolarized mitochondria are unable to recruit Parkin, instead they undergo retrograde transport (Figure 2a) (Lin et al., 2017). This suggests a model where dysfunctional mitochondria are transported retrogradely to ensure a quick response of axonal mitochondria to mild damage; however, when damage is irreversible, the PINK1/Parkin pathway plays a critical role in promoting local mitochondrial degradation through mitophagy.

## 4 CONCLUDING REMARKS

Maintaining a proper mitochondrial trafficking and positioning is crucial to determine the energy gradients throughout the cell. In MEFs, this process seems to be dependent on Miro1 and is essential to promote cell migration (Schuler et al., 2017). In neurons, however, mitochondria play an additional role in Ca^2+^ homeostasis. This implies that most likely more complex mechanisms are in place to properly distribute mitochondria throughout the different neuronal compartments. Although many features of mitochondrial transport are known, there are still several open questions in the field. Future studies that clearly identify which proteins are connecting mitochondria to dynein are required. As important, would be the unveiling of the mechanisms that dictates when mitochondria are bound to kinesin‐1 through the Miro‐TRAK complex or through Syntabulin. This also applies to docking mechanisms, where understanding in which situations mitochondria are preferentially anchored by Syntaphilin or by a myosin. One interesting hypothesis is that these mechanisms differ depending on the compartment. Indeed, mitochondrial transport in dendrites is mainly dependent on dynein and TRAK2, where axonal transport is mediated by kinesin‐1, dynein, and TRAK1. Additionally, actin plays a key role in dendritic spines, making it plausible that myosin‐mediated docking is more relevant at post‐synaptic sites; whereas, Syntaphilin is mainly present in axons and probably more associated with presynaptic docking. These assumptions corroborate with the fact that mitochondria have different functions in pre and post‐synaptic sites. While pre‐synaptic mitochondria are required to buffer Ca^2+^ and avoid extreme states of excitation (Vaccaro, Devine, Higgs, & Kittler, 2017); in post‐synaptic sites mitochondria power plasticity and local protein translation (Rangaraju, Lauterbach, & Schuman, 2019). Future studies should focus on how all these players are involved in mitochondrial transport and docking and which mediate the transport and consequent arrest in each specific neuronal sub‐compartment.

As post‐mitotic cells, neurons require the maintenance of a constant pool of functional mitochondria in each compartment. Thus, an even tighter regulation of mitochondrial quality control mechanisms must be at play. Notably, mitophagy has been recently linked to genetic forms of Parkinson's disease. Therefore, understanding mitochondrial turnover is an important emerging feature in neuronal biology. Whether mitophagy takes place at the synapse or if damaged mitochondria actually travel to the cell body to be degraded, is still somewhat controversial. It is possible that both situations occur depending on the level of mitochondrial damage (Figure 2). If it is only a mild damage, mitochondria are still able to return to the cell body; but if mitochondria are severely dysfunctional, then they most probably undergo local degradation. Additionally, basal mitophagy occurs in vivo, but is independent on PINK1 (McWilliams et al., 2018), which further corroborates that different kinds of damage lead to different quality control mechanisms. Worth highlighting, most of the results reported so far rely on acute depolarizing agents at high concentrations, which may lead to non‐physiological mechanism of mitochondrial degradation. Thus, future studies should address the problem of mitophagy inducers and they should also attempt to recapitulate these results in vivo.

Finally, as different neurons have different functions, it is very likely that neurons also have different thresholds to tolerate intrinsic toxicity, demanding a rapid clearance of the toxic source. Therefore, one could speculate that different types of neurons have different mechanisms to deal with local degradation of damaged mitochondria.

Understanding how mitochondria travel in neurons and what processes occur at synapse constitute key unanswered questions that will open the avenues toward understanding how this organelle is involved in maintaining a functional neuron and, ultimately, a healthy brain. Concomitantly, will also give rise to new insights on the pathophysiological mechanisms of neurodegenerative diseases.

## Data Availability

N/A
